# Association between metabolic syndrome and healthcare work status in Ekiti State, Nigeria

**DOI:** 10.11604/pamj.2021.39.257.26201

**Published:** 2021-08-20

**Authors:** Bolade Folasade Dele-Ojo, Taiwo Hussean Raimi, Joseph Olusesan Fadare, Samuel Ayokunle Dada, Ebenezer Adekunle Ajayi, David Daisi Ajayi, James Ayodele Ogunmodede, Akande Oladimeji Ajayi

**Affiliations:** 1 Department of Medicine, Ekiti State University, Ekiti State University Teaching Hospital, Ado-Ekiti, Ekiti State, Nigeria,; 2 Department of Pharmacology and Therapeutics, Ekiti State University, Ekiti State University Teaching Hospital, Ado-Ekiti, Ekiti State, Nigeria,; 3 Department of Chemical Pathology, Ekiti State University Teaching Hospital, Ado-Ekiti, Ekiti State, Nigeria,; 4 Department of Medicine, University of Ilorin, PMB 1515, Ilorin, Kwara State, Nigeria

**Keywords:** Metabolic syndrome, cardiovascular diseases, night shift, healthcare workers, abdominal obesity, elevated cholesterol

## Abstract

**Introduction:**

metabolic syndrome portends an increased risk of cardiovascular events and death. Evidence showed that healthcare workers are at higher risk of cardiovascular events because of their engagement in night-shift work. Therefore, this study determined the association between metabolic syndrome and healthcare work status in Ekiti State, Nigeria.

**Methods:**

this was a cross-sectional study involving 105 healthcare workers and 143 non-healthcare workers. The diagnosis of metabolic syndrome was made based on the International Diabetic Federation criteria: abdominal obesity plus, any two of: elevated blood pressure ≥ 130/85 mmHg or previous diagnosis of hypertension on the use of antihypertensive medications; impaired fasting glucose; elevated triglycerides; and low HDL-cholesterol. Factors associated with metabolic syndrome were analysed using univariable and multivariable analysis.

**Results:**

men comprised 37.9% of the study population and the mean age was 42.1 ± 9.7 years. The prevalence of metabolic syndrome was similar in both groups (HCWs-29.5% vs non-HCWs- 28.0%, p-value=0.789); overall prevalence was 28.6%. Abdominal obesity, elevated total cholesterol and elevated LDL-cholesterol occurred more frequently in HCWs than in non-HCWs: (68.6% vs 55.2%, p-value=0.034; 65.7% vs 39.2%, p-value= < 0.001 and 50.5 vs 28.7%; p-value < 0.001) respectively. Female sex (aOR: 3.67, 95% CI: 1.74-7.45; p < 0.001) and obesity (aOR: 4.39, 95% CI: 2.31-8.37; p < 0.001) were associated with metabolic syndrome.

**Conclusion:**

a similar prevalence of metabolic syndrome was observed in the healthcare workers and the non- healthcare workers. However, abdominal obesity, elevated total cholesterol and elevated LDL-cholesterol occurred more frequently in healthcare workers than in non- healthcare workers.

## Introduction

Cardiovascular disease (CVD) is the leading cause of mortality, morbidity and disability in Nigeria and the world at large [1]. According to the World Health Organization, 17.9 million people die every year from CVDs [2]. The burden of cardiovascular disease is greater in the low- and middle-income countries (LMICs) [2]. Metabolic syndrome (MetS) is a cluster of metabolic abnormalities that include hypertension, central obesity, insulin resistance and atherogenic dyslipidaemia [3]. It is associated with an increased risk of cardiovascular disease [4, 5]. MetS was previously thought to be rare in Africa, but it is now a major public health concern, with increasing prevalence in African populations [6]. A systematic review showed the prevalence of MetS in sub-Saharan Africa was 18%, it was higher in women and residents of semi-urban areas [7]. The prevalence of MetS in Nigeria has been documented to be as low as 12.1% in a study done in rural communities, to as high as 54.3% in people with diabetes mellitus [8]. The prevalence of MetS is affected by factors such as age, location, sex, and occupation [5, 9].

Certain occupations are at higher risk of MetS [9]. Night shift work is generally associated with persistent abnormal alignment between the endogenous circadian timing system and behaviour cycles, leading to MetS [10]. Adeoye *et al*. reported a prevalence of metabolic syndrome among healthcare workers to be 24.2% [11] Increased shift work duration has also been found to be associated with metabolic syndrome, hypertension, elevated waist circumference and hyperglycaemia [12]. Shift work is associated with risk factors for coronary artery disease (CAD) as it disrupts the normal circadian rhythm [13-15].

Some local studies have been done on the prevalence of MetS in different occupation, however, none has compared the prevalence of MetS among healthcare and non-healthcare workers. Hence, this study determined the association between metabolic syndrome and healthcare work status in Ekiti State, Nigeria. We hypothesize that metabolic syndrome will be more prevalent among healthcare workers than non-healthcare workers.

## Methods

**Study setting and design:** this was a cross-sectional study conducted in two centres in Ado-Ekiti, Ekiti State, Nigeria between August 2018 to December 2018. The participating facilities were Ekiti State University Teaching Hospital (EKSUTH) and Ekiti State University (EKSU). A total of two hundred and forty-eight (248) participants were recruited from both sites over a period of five (5) months. One hundred and five (105) healthcare workers (night shift workers) were recruited from EKSUTH while one hundred and forty-three (143) University staff who were non-healthcare workers were recruited from EKSU, in ratio 1: 1.4.

**Study population:** participants were Staff of EKSUTH and EKSU, aged 18 years and above. Chronically ill patients and pregnant women were excluded from the study. The sample size calculation was based on the estimated prevalence of metabolic syndrome of 24.2% among healthcare workers in a similar study [11] and the prevalence of metabolic syndrome of 8.8% among non-healthcare workers [11]. The required minimum sample size was calculated using the Epi info online calculator, which was 74 for healthcare workers and 103 for non-healthcare workers. However, this was increased to 105 for HCWs and 243 for non-HCWs, taking into account the attrition rate of 10% and also to increase the power of the study.

**Data collection:** baseline clinical and demographic characteristics were obtained from the study participants using a self-administered questionnaire. The measurements of weight, height, waist and hip circumferences and blood pressures were obtained by standard protocols. The weight of participants was taken with light clothing and standing barefooted with the weights measured to the nearest 0.1kg and the height to the nearest 0.1m without cap or head-gear, were determined with bathroom scales and stadiometer respectively. The body mass index (BMI) was calculated from weight (in kilograms) divided by the square of the height (in metres) while the waist-to-hip ratio (WHR) was calculated from the values of waist divided by the hip circumferences.

**Definitions:** classification of body mass index: based on the World Health Organization of body mass index (BMI), BMI is defined thus: underweight < 18.5 kg/m^2^, normal 18.5-24.9 kg/m^2^, overweight 25-29.9kg/m^2^ and obese ≥ 30kg/m^2^ [16].

**Blood pressure (BP):** BP measurement was done following the WHO protocol as thus: each participant was asked to sit quietly and rest for 15 minutes with legs uncrossed and then the BP was measured in the left arm in the sitting position with the aid of Omron Sphygmomanometer and some were taken manually with mercury sphygmomanometer [17]. Two BP measurements were taken three minutes apart; the mean BP was taken as the final BP [17].

**Biochemical analysis:** venous samples for fasting plasma glucose and lipid were obtained after 8-12 hours overnight fast, venous sample were obtained through aseptic techniques. The serum was immediately separated by centrifugation and stored in a freezer at -8 C to await batch analysis in the laboratory. Fasting plasma glucose was done using the glucose oxidase method. Total cholesterol and high-density lipoprotein cholesterol were determined using the cholesterol oxidase and triglyceride levels were determined using the triglyceride reagent. However, low-density lipoprotein cholesterol (LDL-C) was calculated using Friedewald equation [LDL-C (mmol/L) = total cholesterol - (HDL-cholesterol) - (triglyceride/5)] [18].

**Diagnosis of metabolic syndrome:** metabolic syndrome was defined based on the International Federation of diabetes criteria [19]: a) central obesity (defined as waist circumference > 94 cm in men and > 80 cm in women) plus, any two of the following; b) blood pressure ≥ 130/85 mmHg or previous diagnosis of hypertension on the use of antihypertensive medications; c) fasting plasma glucose ≥ 5.6 mmol/L (100 mg/dl) or previously diagnosed type 2 diabetes mellitus; d) triglycerides ≥ 1.7 mmol/L (150 mg/dl); e) HDL-cholesterol < 1.03 mmol/L (40 mg/dl) in men < 1.30 mmol/L (50 mg/dl) in women.

**Statistical analysis:** statistical analysis was performed using the Statistical Package for Social Sciences (SPSS) 23.0 (Chicago Ill). Categorical variables were expressed as frequencies and percentage. The chi-square was used to compare the categorical variables between two groups. Factors associated with metabolic syndrome were analysed using univariate and multivariate analysis in the study population. Variables with a p-value of less than 0.2 in the univariable analysis, were included in the multivariable model. The results were presented as the odds' ratio with corresponding 95% confidence interval. For all tests, statistical significance was set at a p-value < 0.05.

**Ethical considerations:** ethical approval with the reference number-EKSUTH/A67/2018/08/004 was obtained from the Research and Ethics Committee of Ekiti State University Teaching Hospital, Ado-Ekiti. Also, informed written consent was obtained from each participant before the commencement of the study.

## Results

**The demographic, anthropometric and clinical characteristics of the study population:** overall, the study participants were 248 which consisted of 105 (42.3%) healthcare workers and 143 (57.7%) non-healthcare workers. Both HCWs and Non-HCWs were age and sex-matched, as shown in Table 1. The mean age of the study participants was 42.1 ± 9.7 years. There were 94 males (37.9%) and female participants 154 (62.1%). More HCWs had abdominal obesity; elevated total cholesterol and elevated LDL-cholesterol than non-HCWs. Out of healthcare workers, 35 (33.3%) were doctors and 70 (66.7%) were nurses while out of the non-healthcare workers, 15 (10.5%) were academic staff and 128 (89.5%) were the non-academic staff.

**Table 1:** comparison of demographic, anthropometric and biochemical characteristics between healthcare and non-healthcare workers

Variable	Healthcare worker (n = 105)	Non-healthcare worker (n = 143)	Total (N = 248)	p-value
	n (%)	n (%)	N (%)	
**Gender**				0.182
Male	36 (34.3)	61 (42.7)	97 (39.1)	
Female	69 (65.7)	82 (57.3)	151 (60.9)	
**Age (in years)**				0.182
< 45	36 (34.3)	61 (42.7)	97 (39.1)	
≥ 45	69 (65.7)	82 (57.3)	151 (60.9)	
Obesity (BMI ≥ 30kg/m^2^)	35 (33.3)	36 (25.2)	71 (28.6)	0.160
Abdominal Obesity	72 (68.6)	79 (55.2)	151 (60.9)	0.034*
Elevated Systolic Blood Pressure	44 (41.9)	52 (36.4)	96 (38.7)	0.376
Elevated Diastolic Blood Pressure	29 (27.6)	52 (36.4)	81 (32.7)	0.147
Impaired Fasting Plasma Glucose	30 (28.6)	57 (39.9)	87 (35.1)	0.066
Elevated total cholesterol	69 (65.7)	56 (39.2)	125 (50.4)	<0.001*
Elevated triglyceride (mmol/L)	20 (19.0)	40 (28.0)	60 (24.2)	0.105
Low HDL-cholesterol (mmol/L)	17 (16.2)	19 (13.3)	36 (14.5)	0.521
Elevated LDL-cholesterol (mmol/L)	53 (50.5)	41 (28.7)	94 (37.9)	<0.001*

Keys: BMI, body mass index; HDL, high density lipoprotein; LDL, low density lipoprotein; *, statistically significant.

Prevalence of metabolic syndrome: the prevalence of MetS among healthcare workers and non-healthcare workers in this study was similar (29.5% vs 28.0%; p-value= 0.789) respectively, while the overall prevalence in the study population was 28.6%. The characteristics of participants with metabolic syndrome are shown in Table 2.

**Table 2:** characteristics of participants with metabolic syndrome

	Metabolic Syndrome			
Variable	Yes	No	Total	p-value
	n (%)	n (%)	N (%)	
**Gender**				
Male	11 (11.7)	83 (88.3)	94 (100.0)	<0.001*
Female	60 (39.0)	94 (61.0)	154 (100.0)	
**Age (in years)**				
< 45	38 (39.2)	59 (60.8)	97 (100.0)	0.003*
≥ 45	33 (21.9)	118 (78.1)	151 (100.0)	
**Obesity (BMI ≥ 30kg/m^2^)**				
Yes	39 (54.9)	32 (45.1)	71 (100.0)	<0.001*
No	32 (18.1)	145 (81.9)	177 (100.0)	
**Total cholesterol**				
Elevated	41 (32.8)	84 (67.2)	125 (100.0)	0.143
Normal	30 (24.4)	93 (75.6)	123 (100.0)	
**LDL-cholesterol (mmol/L)**				
High	33 (35.1)	61 (64.9)	94 (100.0)	0.078
Normal	38 (24.7)	116 (75.3)	154 (100.0)	
**Family history of hypertension**				
Yes	23 (25.0)	69 (75.0)	92 (100.0)	0.322
No	48 (30.8)	108 (69.2)	156 (100.0)	
**Family history of diabetes**				
Yes	11 (26.8)	30 (73.2)	41 (100.0)	0.780
No	48 (30.8)	108 (69.2)	156 (100.0)	
**Healthcare work status**				
Yes	31 (29.5)	74 (70.5)	105 (100.0)	0.789
No	40 (28.0)	103 (72.0)		

Keys: BMI, body mass index; LDL, low density lipoprotein, *, statistically significant

**Correlates of metabolic syndrome:** as shown in Table 3 below, female sex, age < 45 years and obesity reached significance in the univariable analysis. However, female sex and obesity were the only two factors that were associated with metabolic syndrome in the multivariable analysis. Female participants had about four times likelihood of developing MetS than male counterparts (aOR: 3.67, 95% CI: 1.74-7.45; p = 0.001) and obese individuals also had over four times likelihood of developing MetS than non-obese people (aOR: 4.39, 95% CI: 2.31-8.37; p < 0.001) and were factors associated with metabolic syndrome.

**Table 3:** univariate and multivariate factors associated with metabolic syndrome

	Univariable analysis		Multivariable analysis	
	Odd Ratio (95% CI)	p-value	Adjusted Odd Ratio (95% CI)	p-value
Gender, female	4.82 (2.37 - 9.77)	<0.001**	3.67 (1.74 - 7.45)	0.001*
Age, < 45 years	2.30 (1.31 - 4.04)	0.003**	1.74 (0.93 - 3.28)	0.084
Obesity (BMI ≥ 30kg/m^2^)	5.52 (3.02 - 10.11)	<0.001**	4.39 (2.31 - 8.37)	<0.001*
Elevated total cholesterol	0.66 (0.38 - 1.15)	0.143**	1.13 (0.52 - 2.48)	0.758
Elevated LDL-Cholesterol (mmol/L)	1.65 (0.94 - 2.89)	0.078**	1.45 (0.66 - 3.18)	0.352
Family history of hypertension	0.75 (0.42 - 1.34)	0.322	-	-
Family history of diabetes	0.90 (0.42 - 1.91)	0.780	-	-
Healthcare work status	1.08 (0.62 - 1.88)	0.789	-	-

** , Variables with a p-value of less than 0.2 in the univariable analysis were selected and included into the multivariable model. Keys: CI, confidence interval; BMI, body mass index; LDL, low density lipoprotein, *, statistically significant.

## Discussion

The prevalence of MetS among healthcare workers and non-healthcare workers in this study was similar (29.5% vs 28.0%; p-value= 0.789) respectively, while the overall prevalence in the study population is 28.6%. Significant disparities in the risks of metabolic syndrome by occupation type have been reported, noting that shift workers such as HCWs are at greater risk for the development of the metabolic syndrome [9, 20-22]. The prevalence of MetS syndrome among healthcare workers in this present study (29.5%) is higher than 24.2% that was reported by Adeoye *et al*. in HCWs a few years earlier [11]. In this study, the prevalence is also higher than the prevalence of MetS (20%) that was reported among teachers and bank workers [21]. This trend is suggesting a rising prevalence of MetS over the years, which may be attributable to a parallel rise in the prevalence of obesity [23]. Considering the similar prevalence of MetS among both HCWs and non-HCWs, this study highlights the importance of other factors in the pathogenesis of MetS apart from disturbance of circadian rhythm present in night shift-healthcare workers.

Also, both groups reside in the urban area and are exposed to similar risk factors such as unhealthy diet, physical inactivity and stress. Aside from the hormonal and imbalances of the circadian rhythm, obesity and physical inactivity are the driving force behind the syndrome [3]. Even though HCWs may be disadvantaged because of increased cardiovascular risk associated with their shift job, this may have been negated by the effect of good knowledge of cardiovascular risk factors and practice of precautionary measures to prevent the adverse outcome [24]. Hence, HCWs are not necessarily healthier than non-HCWs, but they have a greater awareness of cardiovascular risk factors and greater access to medical care.

In this study, abdominal obesity, elevated total cholesterol and elevated LDL-cholesterol occurred more frequently in healthcare workers than in non- healthcare workers. A study has documented an increase in the atherogenic index of the plasma score in night shift workers compared to daytime workers [25]. This is in concordance with previous studies that showed that increased shift work duration was associated with elevated waist circumference [12]. A previous study has shown that HCWs have a higher prevalence of hyperlipidaemia than non-HCWs [26].

In our study, hypercholesterolaemia is significantly higher in HCWs than in non-HCWs. This has invariably placed the HCWs at greater risk of atherosclerosis and cardiovascular disease than the non-HCWs. This may be attributable to a sedentary lifestyle, higher socioeconomic status, night shift job schedule, inadequate time for a healthy lifestyle such as regular physical activity and preparation of healthy meals. Noteworthily, elevated total cholesterol and low-density lipoprotein cholesterol have been shown to be important risk factors for coronary artery disease, hypertension and stroke [27]. A systematic review carried out in sub-Saharan Africa has demonstrated the relationship between cardiovascular mortality and elevated plasma cholesterol [28]. High prevalence of cardiovascular risk factors such as hypertension, obesity and hypercholesterolaemia has also been reported among healthcare workers [29, 30].

Furthermore, this study showed that female sex was associated with MetS among the study participants. Sex disparities in the risks of metabolic syndrome have been documented among Nigerian healthcare workers, as women had an increased likelihood of developing MetS compared with men [11]. Increased prevalence of metabolic syndrome among women could be as a result of increased physical inactivity, use of hormonal contraceptives and increased central obesity reported to be common among the women. Indeed, a similar study had earlier reported that women were about eleven times more likely to have central obesity than men [31]. Also, this study showed that MetS is associated with obesity. Individuals with general obesity are more likely to have abdominal obesity, which is an important determinant of MetS.

This study has some limitations as the data are cross-sectional, hence casual inference is weak. Also, this study was carried out at a single geopolitical zone in the country. Having the hypothesis generated from this study tested in other teaching hospitals/universities in Nigeria will increase the strength of the study. Note worthily, the strength of this study is that, it is one of the few studies that has compared the prevalence of metabolic syndrome among healthcare workers and non-healthcare workers in our country.

## Conclusion

A similar prevalence of metabolic syndrome was observed in both the healthcare workers and non- healthcare workers. However, abdominal obesity, elevated total cholesterol and elevated LDL-cholesterol occurred more frequently in healthcare workers than in non- healthcare workers.

### What is known about this topic

- Shift workers such as healthcare workers are at increased risk of developing metabolic syndrome compared with non-shift workers;
- Women are of an increased likelihood of developing metabolic syndrome compared with men.

### What this study adds

- This study reveals an increasing prevalence of metabolic syndrome over the years, which may be attributable to a parallel rise in the prevalence of obesity;
- Considering the similar prevalence of MetS among both healthcare workers and non- healthcare workers, this study highlights the importance of other factors in the pathogenesis of metabolic syndrome apart from disturbance of circadian rhythm present in night shift-healthcare workers;
- This study also shows that healthcare workers might be at greater risk of cardiovascular events because of the presence of other important cardiovascular risk factors such as abdominal obesity, elevated total cholesterol and elevated LDL-cholesterol that occurred more frequently in them when compared with the non- healthcare workers.
